# How the perfectionistic climate of a sports team predicts the athletic performance of elite athletes: a case study of the CUBAL women’s basketball team

**DOI:** 10.3389/fpsyg.2024.1415196

**Published:** 2024-07-31

**Authors:** Meng Meng, Rong-Hai Su, Kohei Kogiso, Rong-Rong Zheng, Lin Chen, Wei Wei, Wei Li, Mao-Chou Hsu

**Affiliations:** ^1^Department of Physical Education, Xiamen University, Xiamen, China; ^2^College of Physical Education and Sports, Beijing Normal University, Beijing, China; ^3^Graduate School of Humanities and Social Sciences, Hiroshima University, Hiroshima, Japan; ^4^School of Physical Education, Education University of Hong Kong, Hong Kong, Hong Kong SAR, China; ^5^Department of Recreation Sports Management, Tajen University, Pingtung, Taiwan

**Keywords:** perfectionistic climate, challenge stressors, threat stressors, coping strategies, athletic performance

## Abstract

**Objective:**

In competitive sports, understanding how the perfectionistic climate within teams influences the performance of elite female athletes can provide valuable insights for enhancing coaching practice and athletic achievement. Based on the cognitive appraisal theory of stress, this study constructs a dual-path model using stressors and coping strategies as mediators, referred to as the Perfectionistic Climate on Athletic Performance model (PCPM). The study explores the predictive role of the perfectionistic climate within sports teams on the athletic performance of elite female basketball players.

**Methods:**

The empirical study the relationships among the variables in the model using a sample of 125 core players from the top-level women’s basketball teams in the 24th CUBAL24 tournament in 2022. A Structural Equation Modeling (SEM) analysis was conducted using AMOS 20.0, primarily employing the bias-corrected Bootstrap method to test the dual-path model.

**Results:**

The findings reveal double-edged paths towards a perfectionistic climate on athletic performance. In the positive pathway, a perfectionistic climate can positively predict athletic performance through challenge-related sources of stress and positive coping strategies. In the negative pathway, a perfectionistic climate can negatively predict athletic performance through threat-related sources of stress and negative coping strategies.

**Conclusion:**

Coaches need to pay attention to athletes’ cognitive evaluations of the perfectionistic climate as a source of pressure. By setting challenging goals, coaches can guide athletes to view the perfectionistic climate of the sports team as a source of challenging pressure, thus unleashing their potential. Coaches should actively guide athletes in coping with the pressure brought about by the perfectionistic climate, enhancing their ability to handle stress. This will enable athletes to better adapt to the team’s perfectionistic climate and further improve individual and team athletic performance.

## 1 Introduction

Achievement Goal Theory posits that the action goals individuals set in specific achievement situations are the result of the interaction between personality traits and social environmental factors (Nicholls, 1984, 1989). Dweck and Leggett (1988) further explain that personality traits are variables that determine the prior probability of individuals setting action goals and engaging in corresponding behavioral patterns, while social environmental factors can subtly change individuals’ action goals and behavioral patterns. Flett et al. (2002), building upon this idea, proposed a Preliminary Model of the Development of Perfectionism, which elaborates on the factors contributing to the formation of individual perfectionism and emphasizes the significant role of life experiences and the social environment in its development. Perfectionism is a tendency to not accept anything less than perfection (Stoll et al., 2008). It is a multidimensional personality trait (Frost et al., 1990) characterized by striving for flawlessness, setting excessively high standards for oneself, and being overly critical of one’s behaviors or overly sensitive to mistakes (Rice and Preusser, 2002). Perfectionism is commonly found among athletes and has a significant impact on their athletic performance (Stoeber, 2011, 2012). Hill and Grugan (2020) introduced the concept of perfectionistic climate, building upon the Achievement Goal Theory and the Preliminary Model of the Development of Perfectionism. Perfectionistic climate refers to the informational cues and goal structure that align with the pursuit of perfect performance and the rejection of any flaws in achievement contexts. It extends the investigation of perfectionism from individual internal personality traits to external social environments. This study draws on previous research (Chen and Gogus, 2008; Hill and Grugan, 2020) and further suggests that a perfectionistic climate reflects the homogenized beliefs and expectations related to perfectionism that team members develop during social interactions.

It is well-known that an athlete’s performance is directly affected by external factors such as climate conditions (Cheng and Zheng, 2019) and competition rules (Li, 2015), in addition to being influenced by innate attributes like genetic factors (Falahati and Arazi, 2019) and muscle fiber composition (Edman et al., 2019), as well as psychological elements such as achievement motivation (Zuber and Conzelmann, 2014) and emotional intelligence (Zhao et al., 2016), which includes team members’ personalities (Beauchamp et al., 2007), and the psychological climate of the sports team. Previous research has shown that the motivational climate of a sports team (Duda, 1992; Philyaw, 2021), collective efficacy (Dithurbide et al., 2009; Muñoz et al., 2023) and team cohesion (López-Gajardo et al., 2023; Oh and Yoo, 2023) can significantly predict athletic performance. In the pursuit of victory, elite sports teams often develop a culture of striving for excellence, which can lead to the formation of a perfectionistic climate within the team. On one hand, this climate may represent a synonym for harsh demands and suppression of individuality; on the other hand, it can reflect an attitude of striving for excellence, serving as a cohesive force that drives the team and motivates continuous self-improvement. This climate can significantly predict athletes’ performance. Elite sports teams often form a culture of pursuing excellence on the path to victory, and the sports team is likely to form an atmosphere of perfectionism. On the one hand, it may be a synonym for demanding perfection and suppressing individuality; on the other hand, it may also be an attitude of the team pursuing excellence, and it is a spiritual force that cohere the team and drives continuous self-transcendence. Previous research has focused on the relationship between individual-level internalized perfectionism and athletic performance (Hill et al., 2014), with some studies affirming that Olympic champions possess perfectionistic traits (Gould et al., 2002) while others view perfectionism as maladaptive and detrimental to performance (Anshel and Mansouri, 2005; Flett and Hewitt, 2005). However, the impact of a perfectionistic climate as an external factor on athletic performance remains unknown.

In the competitive arena, basketball stands out as a sport heavily depends on teamwork and individual skills, captivating not only because of the thrilling moments in the game but also due to the intricate psychology and team culture behind them. When discussing the athletic performance of elite basketball players, they are often admired for their extraordinary skills and remarkable physical abilities. However, rarely delve into a crucial factor: the perfectionistic climate within the team. Team sports are inherently complex social phenomena, and perfectionistic climate, as a crucial aspect of team culture, emphasizes the pursuit of perfection and the fear of failure. This climate permeates every decision and action of the athletes, affecting their mindset, motivation, and performance. A perfectionistic climate leads basketball players to have higher expectations of their performance but also intensifies their fear of failure. This complexity in mindset allows athletes to either excel exceptionally or make repeated mistakes due to excessive pressure during games. In summary, the perfectionistic climate offers a new perspective for predicting athletes’ performance. In competitive sports, where winning is the goal, striving for excellence and ideal performance is a common objective for both coaches and athletes. Understanding how a perfectionistic climate affects athletic performance and under what circumstances it applies can offer valuable insights for coaching practices.

## 2 Theoretical basis and research hypothesis

Previous studies have reported that most athletes are exposed to a demanding environment where coaches expect them to achieve perfect athletic performances in training or competition (Krane et al., 1997; Udry et al., 1997; Lavallee and Robinson, 2007). Athletes often set standards for themselves that they perceive as excessively high or even unrealistic. Examining the perfectionistic climate within the athlete population can provide a better understanding of the overall social and psychological atmosphere and identify heterogeneous situations in athletic performance within a group perfectionistic environment. In training or competition settings, pressure related to perfectionism is pervasive. According to traditional views, pressure sources can induce stress in athletes, leading to a range of emotional, attitudinal, and behavioral responses. Lazarus and Folkman’s (1984) Cognitive Appraisal Theory of Stress further suggests that individuals have different cognitive appraisals and subsequent responses when facing the same pressure source.

The Cognitive Appraisal Theory of Stress, also known as the Transactional Model of Stress, is a stress theory used to explain the cognitive appraisal and coping processes individuals undergo when faced with stressors (Jiang and Wang, 2022). Selye (1976) categorized stress into eustress (positive stress) and distress (negative stress), where distress leads to negative emotions and adverse effects, while eustress generates feelings of happiness or motivation. Building upon this perspective, Cavanaugh et al. (2000) classified stressors into two categories: Challenge Stressors and Threat Stressors (also known as Hindrance Stressors), a categorization supported by several other scholars (Lepine et al., 2005; Podsakoff et al., 2007). Different individuals may perceive the same stressor as an obstacle or a challenge, thereby influencing their coping strategies for managing stressors (Pindek et al., 2019). Many studies have suggested that individuals facing stressors will inevitably have negative reactions. In reality, stressors can elicit both negative and positive reactions from individuals (Lazarus and Folkman, 1984). Previous research has shown that stressors can lead to negative attitudes and behaviors in individuals (Mawritz et al., 2014). However, the Stress Cognitive Appraisal Theory emphasizes that if individuals believe they have sufficient resources to cope with the stressors, they may adopt positive responses to overcome the adverse implications of stressors. Similarly, as a stressor, the perfectionistic climate can evoke either positive or negative responses in athletes depending on their perceived resources to cope with the stress. If athletes believe they have enough resources to cope with the perfectionistic climate, they may adopt positive coping strategies. Conversely, if they perceive the perfectionistic climate as harmful to their well-being and lack adequate resources to cope with it, they may adopt negative coping strategies, thereby affecting their athletic performance.

Based on the above, the present study adopts the Stress Appraisal Theory as the theoretical foundation, and constructs a dual-path theoretical hypothesis model to predict athletes’ performance based on the perfectionistic climate, referred to as the Perfectionistic Climate on athletic Performance model (PCPM). This model aims to explore the potential mechanisms through which the perfectionistic climate influences athletes’ performance, with the goal of reminding coaches to pay attention to athletes’ adaptability to the perfectionistic climate, in order to further enhance individual and team athletic performance.

### 2.1 Chain mediation of challenging stressors on positive coping strategies

Challenge stressors refer to situations in which individuals believe they can overcome the stress and are motivated by the stress to achieve work goals (Jiang and Wang, 2022). Examples of challenge stressors include workload, job demands, and time pressure (Cavanaugh et al., 2000; Lepine et al., 2005; Crawford et al., 2010; LePine et al., 2015). These stressors are evaluated by individuals as pressures that need to be overcome in order to promote personal growth or achieve established goals. The appraisal of challenge stressors enhances individuals’ expectations of achieving high levels of accomplishment, leading to favorable emotional, attitudinal, and behavioral outcomes and facilitating the attainment of implicit goals (Skinner and Brewer, 2002). Lepine et al. (2005) found that athletes who perceive a perfectionistic climate as a challenge stressor may have a higher pursuit of athletic performance. They are more willing to believe that putting in sufficient effort to overcome the pressures associated with growth or achieving established goals will result in satisfying and valuable outcomes. McCauley et al. (1994) demonstrated that individuals are more likely to experience growth in organizations with high levels of challenging job demands. Although challenge stressors can induce feelings of tension and have negative effects on job satisfaction, organizational commitment, or job performance, the evaluation of challenge stressors often generates positive emotions and attitudes (Lazarus and Folkman, 1984; Cavanaugh et al., 2000; Boswell et al., 2004). Individuals may experience happiness or even excitement (Selye, 1956) in response to challenging evaluations, and these positive emotional responses can counterbalance the negative effects of stress (Podsakoff et al., 2007). Challenging job demands are closely associated with higher levels of job satisfaction and engagement (Campion and McClelland, 1991). The Stress Cognitive Appraisal Theory suggests that stressors perceived as challenging can have a positive impact while inducing stress in employees. As long as employees can effectively cope with these challenges, they may achieve higher job performance, richer work experiences, or more advanced work skills (Sacramento et al., 2013), and the same applies to athletes. According to previous research (Lepine et al., 2005; Zhang et al., 2014), stressors perceived as challenging can generate expectations of greater future benefits for athletes, which can motivate them and counteract the negative implications of stress, resulting in higher levels of athletic performance.

Coping refers to the cognitive and behavioral efforts individuals make to manage internal and external demands that exceed their personal resources (Lazarus and Folkman, 1984). “Positive Coping Strategies” typically refer to adaptive and effective ways of dealing with stressors, such as problem-solving, seeking social support, and positive reframing. Stressors can elicit coping responses from individuals (Xie et al., 2019), and both stressor appraisal and coping strategies are integral to this process. According to the cognitive appraisal theory of stress, when external stressors are appraised as challenging, individuals attempt to reduce the impact of the stressors through positive coping strategies. Research by Hulbert-Williams et al. (2013) showed that if the situational demands are high but individuals believe they are within their capabilities and have sufficient resources to cope, they tend to appraise the situation as challenging, leading to positive coping. Similarly, if a perfectionistic climate is appraised as a challenging stressor by athletes, that is, as a pressure that can bring personal benefits or facilitate growth, they may employ positive coping strategies when they have adequate coping resources, aiming to mitigate or eliminate the impact of the stressor and enhance athletic performance.

By engaging in positive behaviors, individuals can reduce the impact of stressors. This can be achieved through external efforts to change or decrease the demands of stressors or through internal efforts to enhance one’s ability to cope with them (Kahn et al., 1964). In the context of sports teams, athletes can adopt positive strategies to modify stressors and reduce their threat. Kahn et al. (1964) referred to this as an environment-directed coping strategy, which involves altering environmental demands, obstacles, resources, or degrees. However, since athletes operate at the lowest level of the organization and must comply with coaches and team arrangements, they often have limited capacity to change the team’s environment, making it challenging to implement environment-directed coping strategies.

On the other hand, athletes can also enhance their own abilities to cope with stressors more effectively. Kahn et al. (1964) called this a self-directed coping strategy. For instance, in a workplace setting, performance pressure and high-performance demands may prompt employees to increase their efforts to achieve performance goals and prove themselves (Mitchell et al., 2018). Similarly, athletes can continuously learn new knowledge and sports skills to foster personal growth, which may instill confidence in facing challenges and coping positively with stressors. Based on the above analysis, the following research hypothesis is proposed:

H1: Perfectionistic climate is positively correlated with challenging stressors, challenging stressors are positively correlated with positive coping strategies, positive coping strategies are positively correlated with athletic performance, and challenging stressors mediate the relationship between perfectionistic climate and athletic performance. In other words, when athletes perceive a perfectionistic climate as challenging stressors, they are more likely to adopt positive coping strategies to enhance athletic performance.

### 2.2 Chain mediation of threat stressors and negative coping strategies

Threat stressors refer to the stressors that individuals perceive as difficult to overcome and as obstacles to achieving their goals (Jiang and Wang, 2022). These stressors are evaluated by individuals as unnecessary pressures that hinder personal growth or the accomplishment of established objectives. Examples of threat stressors include organizational politics, bureaucratic habits, red tape, role conflicts, lack of job security, and career stagnation. These negative stress events are characterized by their uncontrollable and ambiguous nature (Zhang et al., 2018), which can lead individuals to cope in a negative manner. “Negative Coping Strategies” typically refer to maladaptive or ineffective ways of coping, such as avoidance, denial, or substance use. Threat stressors can result in physical and emotional exhaustion, cognitive resource depletion, and decreased motivation among employees (LePine et al., 2004; Probst et al., 2007; Aryee et al., 2009). As a consequence, individuals may adopt passive coping strategies and exhibit a reduced ability to handle situational demands. Rodell and Judge (2009) research also confirmed that threat stressors can lead to feelings of inferiority and withdrawal. When faced with threat stressors, individuals often find it difficult to eliminate the potential external threat through their own efforts, which significantly undermines their self-efficacy (Zhang et al., 2018). They no longer believe that their efforts and learning can improve outcomes (LePine et al., 2004), resulting in negative reactions toward threat stressors. When threat stressors are strong, individuals may have limited autonomy in determining their work content and methods, leading to a low sense of control over their resources and efforts (Cavanaugh et al., 2000). Even if they make efforts to cope with threat stressors, they may struggle to obtain beneficial returns. Threat stressors can significantly deplete an individual’s physiological and emotional energy, making it difficult to sustain the physical demands of work and maintain a positive work state (LePine et al., 2015). This depletion of physical and emotional resources may decrease the perceived availability of resources for athletes, further negatively influencing their athletic performance.

Previous research has analyzed the relationships between challenge stressors and threat stressors with motivation, organizational commitment, turnover intentions, turnover, withdrawal behaviors, and job performance. The results indicate a significant positive correlation between challenge stressors and the aforementioned variables, while a significant negative correlation exists between threat stressors and these variables (Crawford et al., 2010; Bennett et al., 2018; Webster and Adams, 2020). It can be inferred that when athletes perceive a perfectionistic climate as a threat stressor, it may have a negative impact on their performance. Li et al. (2005) pointed out that coping strategies are important mediating variables that connect the outcomes of training and competition with the psychological and physiological responses to stress, exerting a continuous impact on the direction and intensity of psychological changes. Levy et al.’s (2011) study found that coping strategies significantly affect athletic performance and partially mediate the relationship between pre-competition confidence and subjective performance.

Negative coping strategies involves adjusting one’s interpretation of stressors without changing the objective situation, using strategies such as avoidance, distancing, or finding positive value in negative events (Lazarus and Folkman, 1984). For athletes, the stressor of perfectionistic climate is uncontrollable. On one hand, adopting negative coping strategies can provide psychological comfort and compensation (Mawritz et al., 2014). Psychologically distancing oneself from the stressor can reduce its adverse effects (Folkman et al., 1986). For athletes, since it may be challenging to change the perfectionistic climate stressor, they may exhibit tendencies such as psychological withdrawal, training fatigue, and avoidance of training. These behaviors allow athletes to temporarily escape the interference of the perfectionistic climate stressor and find some relief from the pressure brought about by the organizational atmosphere. As these behaviors are relatively covert in the organization, lacking aggressiveness and destructiveness, and do not have severe consequences, they are common coping strategies among athletes.

On the other hand, athletes may engage in destructive behaviors that violate organizational norms and harm the interests of the organization, such as self-sabotage, resistance to training, or threat the achievement of team goals. However, destructive behaviors are likely to be subject to organizational punishments, and thus, they are not commonly observed among athletes as negative coping strategies. Based on the above analysis, the following research hypothesis is proposed:

H2: There is a significant positive correlation between perfectionistic climate and threat stressors, as well as between challenge stressors and negative coping strategies. There is a significant negative correlation between negative coping strategies and athletic performance. Furthermore, threat stressors and negative coping strategies mediate the relationship between perfectionistic climate and athletic performance. In other words, when athletes perceive a perfectionistic climate as a threat stressor, they are more likely to adopt negative coping strategies, further suppressing their athletic performance.

**Figure 1:**
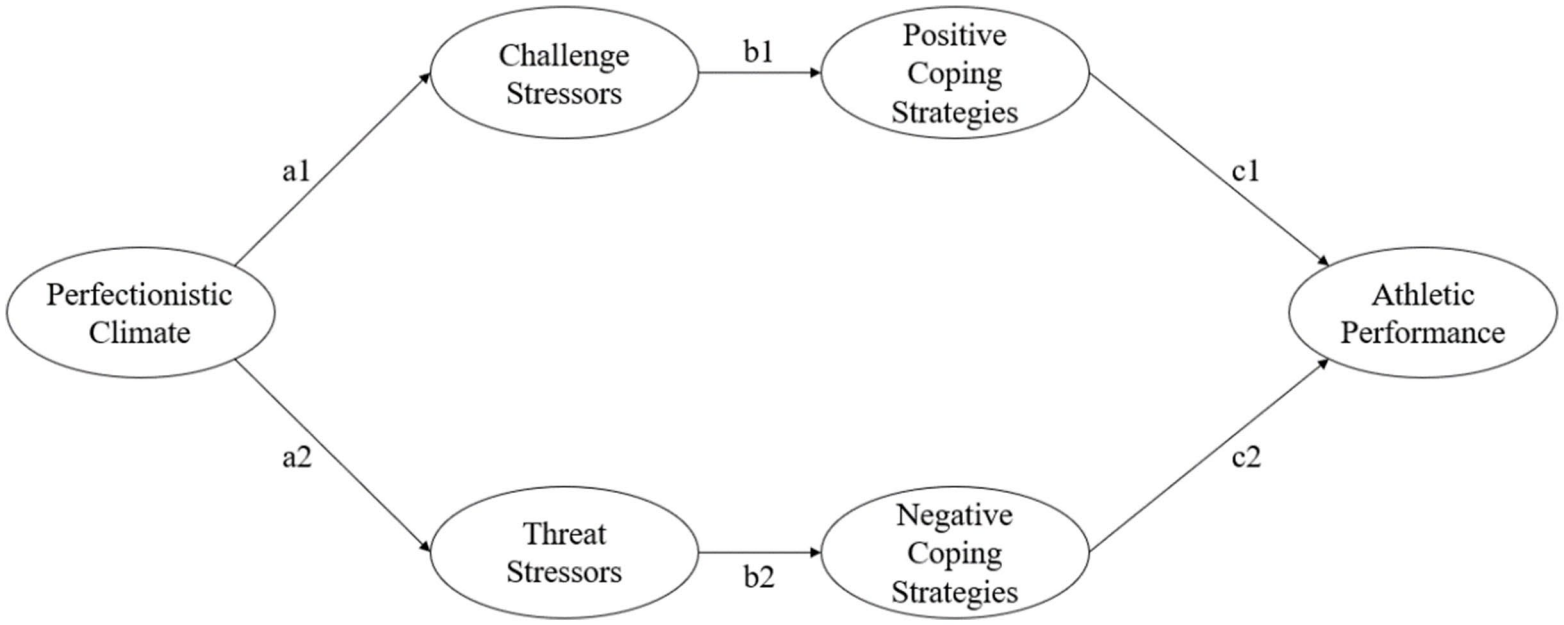
Perfectionistic climate on athletic performance model, PCPM (research hypothesis path: H1 = a1 × b1 × c1; H2 = a2 × b2 × c2).

This study is based on literature to construct a research hypothesis path diagram (Figure 1) on the influence of perfectionist climate on the athletic performance of athletes to explore the dual-path influence mechanism of perfectionist climate on the athletic performance of athletes.

## 3 Research design

### 3.1 Participants

The overlap rate of the roster of women’s basketball high-level sports teams in the top 24 of the Chinese University Basketball Association (CUBAL) over the past 5 years is 87.60%. The top 24 teams in the league exhibit a “pyramid” effect, and the performance of athletes from these teams is sufficiently stable. Previous literature (Su et al., 2022) has indicated the presence of free-riding issues in team cooperation projects. Therefore, it is more reasonable to select core players (operationally defined as the top six players in terms of average points per game within the team) for the study. Based on the aforementioned analysis, this study will focus on the core players from the women’s high-level basketball teams in the top 24 of the 24th CUBAL tournament held in 2022. The Academic Ethics Committee of the School of Physical Education and Sport, Beijing Normal University, approved the research protocol of this study. The inclusion criteria include: (1) being a member of the top 24 high-level women’s basketball teams in the 24th CUBA; (2) being a core player on the team; (3) ranking among the top six in individual average points scored within the team. The exclusion criteria include: (1) players with an average score of zero; (2) players with an average playing time of less than 5 min; (3) players who self-reported injuries during the competition period.

Initially, we consulted the China Student Sports website^1^ to retrieve the list of the top 24 high-level women’s basketball teams and the list of core players for the 24th CUBAL,^2^ and from this information, we established an overall sampling frame. We employed a snowball sampling method triggered by point-to-point cues and contacted target athletes individually through online platforms such as TikTok,^3^ Wotobuy,^4^ and Weibo.^5^ This approach ensured that our study achieved a 100% completion and valid response rate for the questionnaire. The relevant details have been reported in the main text. For this study, we used the semPower package in the R programming language to conduct an *a priori* analysis and determine the minimum sample size required (Moshagen and Bader, 2023). According to the calculated degree of freedom was 398, with the minimum sample size required being over 77. Our study had an effective sample size of 125, which met the minimum sample size requirement. This eliminated the risk of low statistical power due to insufficient sample size. The statistical power of our study was 0.97, which is considered good (Cohen, 1988) as it is above the 0.80 threshold. These results indicate that our study had strong statistical power.

### 3.2 Instrument

#### 3.2.1 Perfectionistic climate

The Perfectionistic Climate Questionnaire-Sport (PCQ-S) was adapted from the study conducted by Grugan et al. (2021). The questionnaire was measured using a 7-point Likert scale and comprised five constructs: Expectations, Criticism, Control, Conditional Regard, and Anxiousness. Each construct consisted of five items. The items went through a process of translation, refinement, and back-translation, resulting in a Chinese version of the questionnaire with a total of 20 items, such as “The coach expects us to perform perfectly on the field or in training” and “Even for small mistakes, the coach criticizes us.” The original scale’s McDonald’s Omega (ω) ranges from 0.82 to 0.86, with expectations = 0.82 (95% CI range = 0.77 to 0.85); criticism = 0.85 (95% CI range = 0.81 to 0.88); control = 0.84 (95% CI range = 0.80 to 0.88); conditional regard = 0.86 (95% CI range = 0.82 to 0.89); anxiousness = 0.84 (95% CI range = 0.80 to 0.87). The factor loadings of the first-order model range from 0.67 to 0.92, indicating that the original scale reportedly possesses good reliability and validity.

#### 3.2.2 Stressors

The measurement method for stressors in this study was based on the research reports of Cavanaugh et al. (2000) and LePine et al. (2004), and adapted from the Task Specific Stressor Scale (TSS) developed by LePine et al. (2015), modified to fit the sports context. Each item underwent translation, refinement, and back-translation processes, resulting in a Chinese version of the scale comprising a total of 16 items, such as “I take training or competition tasks seriously and make efforts to complete them” and “I feel that the resources needed to complete training or competition tasks are insufficient.” The scale was measured using a 7-point Likert scale. Among the items, 10 items measured challenge stressors (CS), including time pressure, task complexity, responsibility, and obligations as job demands, while 6 items measured threat stressors (TS), including task ambiguity, lack of resources, and interpersonal conflicts as job demands. The Confirmatory Factor Analysis (CFA) of the original scale yielded the following results: χ^2^ = 698.29, df = 293, CFI = 0.91, RMSEA = 0.09, SRMR = 0.09, indicating structural validity of the original scale.

#### 3.2.3 Coping styles

The coping styles in this study were based on the Simplified Coping Style Questionnaire (SCSQ) by Xie (1998) in its Chinese version. The questionnaire was adapted and revised to fit the sports context, resulting in a simplified version consisting of 15 items. Examples of the items include “I tend to see the positive side of training or competition outcomes” and “I often fantasize about a miracle happening to change the bad situation.” The Likert 7-point scale was used for measurement. Among the items, 11 items measured Positive Coping Strategies (PCS) and 4 items measured Negative Coping Strategies (NCS). The original scale demonstrated reliability and validity, with a Cronbach’s α of 0.89 for the internal consistency of the positive coping strategy subscale and a Cronbach’s α of 0.78 for the internal consistency of the negative coping strategy subscale. The factor loading values for the first-order model ranged from 0.32 to 0.77, indicate reliability and validity of the original questionnaire.

#### 3.2.4 Athletic performance

Python language was used to extract relevant indicators of the performance of 125 core players from 24 high-level women’s basketball teams in the 24th CUBAL (China University Basketball Association) tournament, held from July 14th to July 21st, 2022, through the Game-Log of WeChat Mini Program. The extracted indicators included Personal Total Score (PTS), Minutes (MIN), Rebounds (REB), Offensive Rebounds (OFF), Defensive Rebounds (DFE), Assists (AST), Steals (STL), Blocks (BLK), Field Goal Made (FGM), Three-point Made (3 PM), Free Throw Made (FTM), Turnovers (TO), Fouls (F), and Technical Fouls (TF), totaling 14 indicators. This study conducted reliability and validity tests on the measurement model of athletic performance. The results showed that McDonald’s Omega (ω) was 0.83, which met the criteria provided by Peters (2014) for reliability testing, indicating internal consistency of the athletic performance measurement model. Based on factor loading values and Modification Indices (MI), some indicators of athletic performance were removed. In the end, 6 indicators were retained: Personal Total Score (PTS), Minutes (MIN), Rebounds (REB), Steals (STL), Field Goal Made (FGM), and Free Throw Made (FTM). The factor loadings of the revised one-factor model ranged from 0.68 to 0.93. The Composite Reliability (CR) was 0.87, and the Average Variance Extracted (AVE) was 0.57, meeting the criteria provided by Zhang et al. (2020) for convergent validity, indicating convergent validity of the revised indicators.

To test the content validity of athletic performance, a questionnaire was administered to 6 experts (3 high-level basketball coaches and 3 professors in the field) to evaluate the representativeness, rationality, and effectiveness of the 6 indicators. More than half of the experts believed that the Minutes (MIN) may not be positively correlated with athletic performance, and it was deemed unreasonable and therefore excluded from the evaluation. According to the formula provided by Jiang et al. (2011) for expert evaluation, the comprehensive evaluation result was R = 86.74, indicating overall expert evaluation. Additionally, based on the suggestions of two basketball coaches, this study adopted the inverse of the athlete’s sports level (ranging from 1 to 5, representing International Elite, National Elite, National Level-1, National Level-2, and no level) to represent the athlete’s contribution to the team. These values were multiplied by the weight coefficients and applied to the calculation of athletic performance in relation to 5 indicators, thereby taking into account both the athlete’s on-court performance and their performance within the team. This approach yielded more objective and realistic data. The mean score was 6.29 ± 5.23 points, the mean number of rebounds was 3.91 ± 2.58, the mean number of steals was 0.89 ± 0.78, the mean field goal percentage was 0.30 ± 0.17, and the mean free throw percentage was 0.23 ± 0.23.

### 3.3 Analysis

Due to the sample’s self-reported nature, missing values were often present. Statistical analysis revealed that the variable had 0 missing values and 3 (2.40%) incomplete cases. The missing rate was relatively small. The missing data imputation method used in this study was the Expectation Maximization (EM) Imputation. The EM imputation method assumes that the missing data is of the missing at random (MAR) type, and uses the current data information and a specified model to perform the “best guess” for the missing data (Bennett, 2001). This method is suitable for continuous variables. Descriptive statistics, correlation analysis, and structural equation modeling (SEM) were conducted using SPSS 27.0 and AMOS 20.0. The relationship between perfectionistic climate, sources of stress, coping strategies, and athletic performance was examined using Pearson correlation analysis, with *p*-values adjusted for multiple comparisons using the false discovery rate (FDR). There were no missing data in this study. In structural equation modeling (SEM), we aim to reveal linear relationships between variables through the covariance matrix. The accurate estimation of the covariance matrix relies on the data normality. If the data does not follow a normal distribution, it can lead to biased estimates of relationships between variables. Therefore, this study analyzes skewness and kurtosis and assesses whether the data meets the criteria for normal distribution based on the standards outlined by Kline (2016).

The majority of the constructs in this study were measured using self-report data, which may introduce common method bias due to the social desirability effect. Therefore, it is necessary to test for common method bias in the model. Given the large number of items involved in the Perfectionistic Climate Questionnaire-Sport, this study followed the approach proposed by Wu and Wen (2011). The items were first parceled based on the five constructs of the Perfectionistic Climate Questionnaire-Sport, using the latent variable constructs as new indicators. The measurement model of the perfectionistic climate was then simplified using item parcels, and the performance data were standardized before analysis. SEM competes the modeling process through a series of assumptions and constraints. It explores the correlations or causal relationships between variables. By analyzing correlations among variables, we gain initial insights into their underlying connections and validate the logical coherence of the hypothesized model. Furthermore, conducting validity and reliability analyses is crucial prior to applying SEM. Rigorous assessment of validity and reliability ensures the collected data is dependable and effective, thereby establishing a robust foundation for SEM. Building on this foundation, SEM allows for deeper exploration of relationships between variables, revealing scientific patterns latent within the data and offering robust guidance for practical applications.

In SEM analysis, indices such as χ^2^/*df*, GFI, AGFI, CFI, NFI, and RMSEA were used to assess model fit: (1) χ^2^/*df* should be less than 2; (2) GFI and AGFI should be greater than 0.90; (3) CFI and NFI should be greater than 0.95; (4) RMSEA should be less than 0.06 (Hu and Bentler, 1999; Kline, 2016). Bootstrapping has more statistical power than causal inference and product of coefficients methods for testing indirect effects (Williams and MacKinnon, 2008; Su et al., 2022). Therefore, this study used bootstrapping to test the chain mediation effects of the dual-path model. The significance of the mediating effects was tested using bias-corrected bootstrap methods. Bootstrap resampling was conducted 5,000 times, and if the 95% confidence interval of the bootstrap did not include 0, the parameter estimate was considered significant; otherwise, it was deemed nonsignificant (Preacher and Hayes, 2008). The significance level for all hypothesis tests was set at *α* < 0.05.

Considering that the 125 female basketball starters were nested within 24 high-level teams, a two-level nested data structure was formed. Specifically, the athletes represented the individual level, and the teams represented the group level. Nested data structures can violate the independence assumption at the individual level, potentially leading to interdependence among athletes’ perfectionism climate scores within the same team. To examine the independence of the individual-level data, this study applied SPSS 27.0 and HLM 6.08 (Hierarchical Linear Modeling) software to conduct Hierarchical Linear Growth Models analysis and obtained the intraclass correlation coefficients (ICC). The nesting effect (also known as the design effect, Deff) was calculated using the formula 1 + (m − 1) × ICC, where m represents the average cluster size. The result showed that Deff = 1.002 < 2, indicating that the nesting effect can be ignored (Muthén and Satorra, 1995).

## 4 Results

### 4.1 Descriptive statistics

An analysis of the study sample basic information is presented in Table 1. The survey covered 125 core players from high-level CUBAL women’s basketball teams, with an average age of 22.02 ± 1.49 years and an average training period of 8.68 ± 2.21 years. Among them, 43 were national second-level athletes, accounting for 34.40%; 67 were national first-level athletes, representing 53.60%; and 15 were master athletes, making up 12.00%. All athletes were undergraduates: 16 were freshmen, accounting for 12.8%; 26 were sophomores, representing 20.8%; 37 were juniors, making up 29.6%; and 46 were seniors, accounting for 36.8%. The average points scored were 6.29 ± 5.23, average rebounds were 3.91 ± 2.58, average steals were 0.89 ± 0.78, average field goal percentage was 0.30 ± 0.17, and average free throw percentage was 0.23 ± 0.23.

**Table 1:** Analysis of research sample basic information.

Team position	Number	Percentage	Athletic level/ grade	Number	Percentage
Point guard	18	14.4	Master of sports	15	12.0
Shooting guard	10	8.0	National first level	67	53.6
Small forward	26	20.8	National second level	43	34.4
Power forward	19	15.2	Freshman	16	12.8
Center	32	25.6	Sophomore	26	20.8
Guard	12	9.6	Junior	37	29.6
Forward	8	6.4	Senior	46	36.8

### 4.2 Common method bias and correlation analysis

A nested competitive model was used to assess the presence of the Common Method Bias. The single-factor Confirmatory Factor Analysis (CFA) yielded χ^2^ = 1619.91, *df* = 405, and the multi-factor CFA yielded χ^2^ = 707.26, *df* = 390. The difference in degrees of freedom (Δ*df*) between the two models was 15, with Δχ^2^ of 912.65 (*p* < 0.001). Thus, this study is not affected by Common Method Bias. As can be seen in Table 2, the absolute values of Skewness in this study range from 0.19 to 0.66, less than 2.00, and the absolute values of Kurtosis range from 0.12 to 1.27, less than 8.00, meeting the testing standards provided by Kline (2016), thus the data in this study can be considered normally distributed. The mean scores of five variables, including perfectionistic climate, sources of stress, and coping methods, range from 3.55 to 5.38, indicating that female basketball players generally have a positive evaluation of the perfectionistic climate, sources of stress, and coping methods. Furthermore, athletic performance is not significantly correlated with perfectionistic climate, sources of stress, and positive coping methods, but it has a significant negative correlation with negative coping methods (r = −0.26, *p* < 0.001); significant correlations exist between the rest of the variables with r values ranging from 0.18 to 0.58.

**Table 2:** Correlation analysis.

	Skew	Kurtosis	M	SD	1	2	3	4	5
1. Perfectionistic climate	0.19	0.61	4.43	1.11	1				
2. Challenge stressors	−0.66	1.27	5.12	1.06	0.43***				
3. Threat stressors	0.53	−0.12	3.55	1.44	0.58***	0.43***			
4. Positive coping strategies	−0.29	1.57	5.38	0.83	0.21*	0.55***	0.29***		
5. Negative coping strategies	−0.33	0.26	4.42	1.27	0.32***	0.28***	0.45***	0.18*	
6. Athletic performance	0.24	−0.59	-	-	−0.04	0.06	−0.04	0.15	−0.26***

*p < 0.05; ***p < 0.001.

### 4.3 Reliability and validity analysis

Reliability and validity tests were conducted on six variables: perfectionistic climate, challenge stressors, threat stressors, positive coping, negative coping, and athletic performance. The analysis results are presented in Table 3. Cronbach’s *α* coefficients are all above 0.70, Composite Reliability (CR) values exceed 0.60, and the Corrected Item-Total Correlation (CITC) for each measurement item or indicator are greater than 0.3, indicating that the scale has good reliability (Churchill and Peter, 1984; Hair, 1998; Zhang et al., 2020). In the confirmatory factor analysis, the standardized factor loadings for each measurement item are greater than 0.50, and the calculated Average Variance Extracted (AVE) values for each latent variable are above 0.50, indicating good convergent validity for the latent variables. Moreover, the square roots of the AVE for each latent variable are greater than the correlation coefficients between variables, indicating good discriminant validity for the latent variables (Fornell and Larcker, 1981). A two-way random effects model was used to analyze the intraclass correlation coefficient (ICC), which was 0.77 (95% CI [0.28, 0.46]), and the ICC was greater than 0.75, which, according to the standard set by Pan and Ni (1999), indicates a high degree of consistency in the measurement of the scales of the present study. In summary, the variables selected in this study exhibit good reliability and validity.

**Table 3:** Reliability and validity analysis.

Variable	Item	Significance of parameter test				Reliability			Validity		
						Corrected item total correlation	Item reliability	Internal consistency	Composite reliability	Factor loadings	Average variance extracted
		Unstd.	Standard error	Z	*p*-value	CITC	SMC	Cronbach’s *α*	CR	Std.	AVE
Perfectionistic climate	EXC1	1.000				0.593	0.448	0.814	0.819	0.669	0.534
	EXC2	1.142	0.177	6.448	***	0.498	0.696			0.834	
	EXC3	1.130	0.189	5.978	***	0.533	0.576			0.759	
	EXC4	0.742	0.145	5.117	***	0.534	0.417			0.646	
	CRI1	1.000				0.621	0.401	0.835	0.844	0.633	0.579
	CRI2	1.161	0.159	7.288	***	0.701	0.719			0.848	
	CRI3	1.167	0.159	7.330	***	0.643	0.746			0.864	
	CRI4	0.932	0.150	6.211	***	0.620	0.449			0.670	
	ConT1	1.000				0.662	0.656	0.903	0.906	0.810	0.710
	ConT2	1.093	0.091	12.038	***	0.670	0.803			0.896	
	ConT3	0.758	0.089	8.549	***	0.576	0.490			0.700	
	ConT4	1.148	0.090	12.710	***	0.637	0.889			0.943	
	ANX4	1.000				0.642	0.491	0.853	0.854	0.701	0.598
	ANX3	0.823	0.124	6.651	***	0.589	0.417			0.646	
	ANX2	1.276	0.148	8.645	***	0.576	0.823			0.907	
	ANX1	1.126	0.137	8.224	***	0.583	0.661			0.813	
	ConR4	1.000				0.717	0.537	0.829	0.836	0.733	0.565
	ConR3	1.134	0.131	8.634	***	0.584	0.806			0.898	
	ConR2	0.866	0.112	7.769	***	0.580	0.545			0.738	
	ConR1	0.940	0.147	6.419	***	0.617	0.372			0.610	
Challenge stressors	CS1	1.000				0.532	0.434	0.892	0.893	0.659	0.583
	CS2	1.361	0.179	7.601	***	0.621	0.638			0.799	
	CS3	1.191	0.161	7.393	***	0.555	0.596			0.772	
	CS4	1.481	0.190	7.781	***	0.553	0.679			0.824	
	CS5	1.221	0.162	7.545	***	0.557	0.627			0.792	
	CS6	0.994	0.142	7.010	***	0.547	0.523			0.723	
Threat stressors	HS1	1.000				0.529	0.384	0.840	0.845	0.620	0.582
	HS2	1.144	0.171	6.681	***	0.650	0.569			0.754	
	HS3	1.361	0.188	7.222	***	0.584	0.808			0.899	
	HS4	1.247	0.187	6.671	***	0.520	0.566			0.752	
Positive coping strategies	PCS1	1.000				0.571	0.263	0.835	0.853	0.513	0.498
	PCS2	1.186	0.245	4.842	***	0.663	0.365			0.604	
	PCS3	1.158	0.209	5.533	***	0.662	0.610			0.781	
	PCS4	1.065	0.196	5.441	***	0.645	0.566			0.752	
	PCS5	1.250	0.221	5.668	***	0.598	0.687			0.829	
	PCS6	1.197	0.227	5.269	***	0.582	0.496			0.704	
Negative coping strategies	NCS1	1.000				0.591	0.462	0.744	0.801	0.680	0.503
	NCS2	1.081	0.206	5.256	***	0.688	0.551			0.742	
	NCS3	1.151	0.222	5.190	***	0.669	0.563			0.750	
	NCS4	0.816	0.184	4.429	***	0.622	0.436			0.660	
Athletic performance	PTS	1.000				0.543	0.863	0.866	0.869	0.929	0.574
	FGM	0.026	0.003	9.705	***	0.540	0.533			0.730	
	FTM	0.033	0.004	9.064	***	0.538	0.486			0.697	
	REB	0.386	0.040	9.620	***	0.527	0.527			0.726	
	STL	0.110	0.013	8.705	***	0.607	0.460			0.678	

***p < 0.001.

### 4.4 Chain mediation analysis of the model

As shown in Table 4, the results indicate that there is significant indirect effect between perfectionistic climate and challenging stressors, with Z value of 3.01 (σ = 0.14) and 95% CI [0.16, 0.69], *p* < 0.001. Similarly, there is significant indirect effect between challenging stressors and positive coping strategies, with Z value of 2.95 (σ = 0.17) and 95% CI [0.25, 0.93], *p* < 0.001. Furthermore, there is significant indirect effect between positive coping strategies and athletic performance, with Z value of 2.03 (σ = 0.16) and CI [0.04, 0.68], *p* = 0.03 < 0.05. The chain mediation effect between challenging stressors and positive coping strategies in the relationship between perfectionistic climate and athletic performance is also significant, with Z value of 1.86 (σ = 0.04) and 95% CI [0.01, 0.17], *p* = 0.01 < 0.05, with effect size υ = 0.08. According to the criteria set by Fern and Monroe (1996) as well as Zheng et al. (2011), this chain mediation exhibits a moderate effect size. In summary, challenging stressors mediate the relationship between perfectionistic climate and athletic performance through positive coping strategies. Additionally, there are significant positive correlations between perfectionistic climate and challenging stressors, challenging stressors and positive coping strategies, and positive coping strategies and athletic performance, supporting research hypothesis H1.

**Table 4:** Results of the chain mediation test of the dual-path model.

	Point estimation	Product of coefficients		95% CI		*p*-value
		σ	Z	Lower	Upper	
**Path**						
a1	0.42	0.14	3.01	0.16	0.69	***
b1	0.51	0.17	2.95	0.25	0.93	***
c1	0.32	0.16	2.03	0.04	0.68	0.03
a2	0.90	0.18	5.01	0.57	1.28	***
b2	0.36	0.11	3.32	0.17	0.60	***
c2	−0.39	0.18	−2.17	−0.81	−0.15	***
**Hypothesis**						
H1	0.07	0.04	1.86	0.01	0.17	0.01
H2	−0.13	0.07	−1.75	−0.38	−0.04	***

Bootstrap 5000; ***p < 0.001.

There is significant positive correlation between perfectionistic climate and threat stressors, with Z value of 5.01 (σ = 0.18) and 95% CI [0.57, 1.28], *p* < 0.001. Similarly, there is significant positive correlation between threat stressors and negative coping strategies, with Z value of 3.32 (σ = 0.11) and 95% CI [0.17, 0.60], *p* < 0.001. On the other hand, there is significant negative correlation between negative coping strategies and athletic performance, with Z value of −2.17 (σ = 0.18) and 95% CI [−0.81, −0.15], *p* < 0.001. The chain mediation effect between threat stressors and negative coping strategies in the relationship between perfectionistic climate and athletic performance is also significant, with Z value of −1.75 (σ = 0.07) and 95% CI [−0.38, −0.04], *p* < 0.001, with effect size υ = 0.14. According to the standards set by Fern and Monroe (1996) and Zheng et al. (2011), this chained mediation demonstrates a moderate effect size. Based on the above, it can be concluded that there is significant positive correlation between perfectionistic climate and threat stressors, significant positive correlation between challenge stressors and negative coping strategies, significant negative correlation between positive coping strategies and athletic performance. Moreover, threat stressors and negative coping strategies play chain-mediated role between perfectionistic climate and athletic performance. Therefore, research hypothesis H2 is supported.

**Figure 2:**
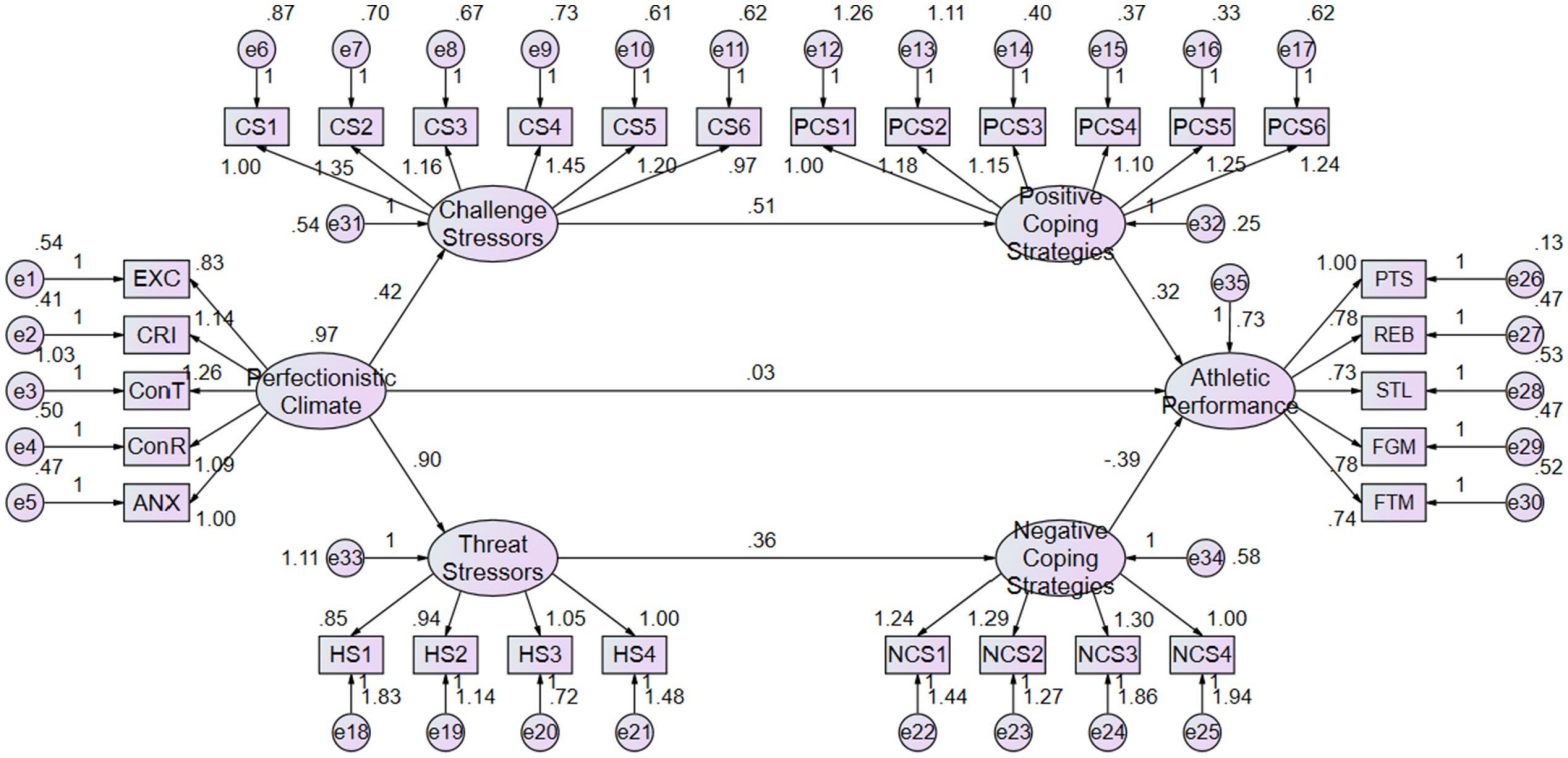
Chain-mediated non-standardized test chart of the dual-path model.

There is no significant correlation between perfectionistic climate and athletic performance, with Z value of 0.23 (σ = 0.13) and 95% CI [−0.19, 0.32], *p* = 0.80. This indicates that there is no significant relationship between perfectionistic climate and athletic performance. The dual-path model is a fully mediated model (Figure 2). The model chi-square value can be impacted by sample size, leading to poorer model fit. To address this, the Bollen-Stine p Correction method can be used to adjust the model fit (Zhang et al., 2020). After applying the Bollen-Stine p Correction (Bootstrap 5000), the probability of a significant discrepancy model is 0.027, which is less than 0.05, indicating that the model fit is affected by the sample size. The adjusted model fit indices are as follows: χ2 = 529.03, χ2/*df* = 1.33, GFI = 0.93, AGFI = 0.94, CFI = 0.95, NFI = 0.95, RMSEA = 0.05. These indices indicate a good fit for the mediated model.

## 5 Discussion

As a team sport, basketball fosters a team atmosphere vastly different from individual sports focused on personal achievements. While individual skills are crucial in basketball, they are not the sole determinant. Basketball relies on teamwork, where each player contributes unique strengths and roles. These diverse roles collectively form a cohesive unit, highlighting the dual nature of perfectionistic within this team environment. This study posits that whether a perfectionistic climate acts as a facilitator or a barrier to athletic performance largely depends on athletes’ cognitive appraisal of stress sources and their coping strategies. The perfectionistic climate on athletic performance has positive and negative predictions. In the positive pathway, a perfectionistic climate can positively predict athletic performance through challenge-related sources of stress and positive coping strategies. Conversely, in the negative pathway, a perfectionistic climate can negatively predict athletic performance through threat-related sources of stress and negative coping strategies.

### 5.1 Interpretation of the positive pathway in the dual-pathway model

Previous scholars (Kahn and Byosiere, 1992; Gilboa et al., 2008) generally believed that sources of stress have predominantly negative effects. However, the empirical results of this study indicate that a perfectionistic climate, as a source of stress, can also have a positive effect on athletic performance if approached with positive coping strategies. The Cognitive Appraisal Theory of Stress particularly emphasizes the individual’s cognitive appraisal of stress sources. During the cognitive appraisal stage, individuals subjectively evaluate the potential benefits and losses of a single stress source (Folkman et al., 1986; Hanton et al., 2012) and assess the resources they perceive as available to cope with the stress source (Lazarus and Folkman, 1984). The team environment, by creating work demands and shaping social interaction patterns, can significantly predict the individual’s assessment of the stress level of a situation and their response to stress (Bliese and Halverson, 1996; Jiang and Probst, 2016). If athletes believe that the perfectionistic climate of their team can bring potential benefits and that they have sufficient resources to cope, they are more likely to have a positive evaluation of the perfectionistic climate as a stress source. Consequently, they may view the perfectionistic climate as a challenge stressor, thereby eliciting more positive coping responses.

Previous research (Haney and Long, 1995; Smith and Christensen, 1995; Calmeiro et al., 2010, 2014; Gaudreau et al., 2010; Nicholls et al., 2010, 2012; Doron and Gaudreau, 2014; Doron and Martinent, 2016) assessing athletic performance through objective or subjective indicators has found a significant correlation between coping strategies and athletic performance. Specifically, positive coping strategies are significantly positively correlated with athletic performance, whereas negative coping strategies are significantly negatively correlated, which is consistent with the findings of this study. And both the positive and negative pathways exhibit moderate effect sizes (Fern and Monroe, 1996; Zheng et al., 2011). When individuals perceive a source of stress as controllable and believe that effort can change the external stress environment, they tend to adopt positive coping strategies. In the Chinese context, organizational discipline within training or competition settings is highly valued, and athletes are expected to obey commands, follow leadership, and consciously adapt to the team atmosphere. Learning to effectively cope with stress sources by enhancing personal capabilities is crucial for improving athletic performance. Lazarus (1993) emphasized that stress and coping strategies interact; stress can stimulate and disrupt coping strategies, while positive coping strategies can reduce the stress perceived from stress sources (Folkman and Lazarus, 1988). When athletes believe they have sufficient abilities and resources to overcome stress sources, the positive pathway towards the perfectionistic climate dual model becomes evident, achieving a new balance between situational demands and individual coping resources. Athletes were better adapt to the perfectionistic climate of the team to meet training and competition requirements. When accustomed to viewing stress sources as challenge stressors, athletes exhibit more positive emotions, more flexible and creative thinking, and are more likely to positively cope with the stress induced by stress sources (Fredrickson, 2001), which also enhances their focus on current training or competition tasks and further improves athletic performance (Gaudreau et al., 2010). Female basketball players of high-level teams who enhance their performance through the positive pathway appear to possess stronger psychological self-regulation capabilities and are better adapted to the perfectionistic climate of the sports team.

### 5.2 Interpretation of the negative pathway in the dual-pathway model

Competitive sports aim to pursue excellent performance, and the perfectionistic climate as a source of stress is pervasive in training and competition environments, increasing emotional exhaustion and negative physical symptoms in some athletes, leading to decreased training satisfaction and training burnout (Nicholls et al., 2012). According to the research views of Wang et al. (2021), Li and Guo (2021) and Yuan (2022), task-related stress sources enhance athletes’ perception of stress, resulting in corresponding attitudes and behaviors. When athletes perceive stress sources as insurmountable and obstructive to their goals, they are more likely to view the perfectionistic climate as a threat stressor. When athletes consider sources of stress uncontrollable and themselves incapable of changing the external environment, they tend to adopt negative coping strategies, through which the perfectionistic climate affects athletic performance via the negative pathway. The perfectionistic climate emphasizes harsh, unreasonable, or even ruthless excessive criticism or punishment of athletes for minor mistakes, or sets rigid and unrealistic expectations, along with stingy recognition or rewards (Grugan et al., 2021). Negative coping is often emotion-centered, manifesting as individuals reducing their negative emotions through avoidance, denial, and other coping strategies (Di et al., 2015). Specifically, it involves efforts to reduce negative emotions cognitively, using avoidance, distancing, or finding positive values in negative events to adjust one’s interpretation of stress sources without changing the objective situation (Lazarus and Folkman, 1984). Therefore, athletes who adopt negative coping strategies towards stress sources psychologically distance themselves from the stress sources to mitigate their adverse impacts, showing low enthusiasm for training, negative attitudes, or even avoidance of training.

When athletes face a culture of perfectionism, they may autonomously and continuously adjust their behaviors and cognitions to regulate the affects of this perfectionistic culture within the sports team. When athletes perceive the culture of perfectionism as a stressful event and adopt negative strategies to cope with the pressure, activating the negative towards the avoidant pathway, they struggle to adapt to the perfectionistic culture of the team, hindering their athletic performance. Avoidance, social withdrawal, self-pity, and self-blame, among other negative coping strategies, may temporarily shield athletes from the pressure of the perfectionistic culture and provide brief relief. However, when individuals fail to take proactive coping actions and instead attempt to change uncontrollable environments or resort to avoidance in response to sources of stress, it typically leads to more negative emotions (Crocker and Graham, 1995; Ntoumanis et al., 2015). The emergence of these negative emotions is mainly due to a lack of control over the stressful situation or a lack of direction towards taking direct coping actions (Ntoumanis and Biddle, 1998). Emotion-focused coping strategies tend to result in adverse outcomes (Crocker and Graham, 1995; Ntoumanis and Biddle, 1998; Ntoumanis et al., 1999; Nicholls and Polman, 2007). Coaches need to be particularly vigilant about overt manifestations of negative coping, such as engaging in destructive behaviors that violate organizational norms and undermine organizational interests (Mawritz et al., 2014). Athletes may retaliate against the organization by skipping training sessions or even boycotting them. Literature on competitive sports indicates a significant negative correlation between negative emotions and athletic performance (Lazarus, 1999; Craft et al., 2003; Woodman and Hardy, 2003; Jones et al., 2005), which also effectively explains the negative impact of negative coping strategies on the performance of elite female basketball players.

### 5.3 The potential pathways through which a perfectionistic affects athletes’ performance

Competitive sports often entail pressure, and in the context of a sports team, the perfectionistic climate arises from social interactions between coaches and athletes, as well as among teammates. It represents the extent to which the team environment affects individuals’ pursuit of perfect athletic performance, constituting a social stressor. Similar to transformational and empowering leadership styles, as well as high-performance expectations from leaders, these social stressors also exhibit double-edged path predictions. Many studies suggest that these factors can trigger negative emotions, leading to emotional exhaustion, deviant behavior, and interpersonal mistreatment (Jahanzeb and Fatima, 2018; Webster et al., 2018; Jiang et al., 2021; Rosen et al., 2021; Venz and Nesher Shoshan, 2021). However, other research demonstrates that employees may perceive these factors as benign pressure and respond positively to achieve favorable outcomes (Majeed and Naseer, 2021). Similar to the above social stressors, the perfectionistic climate can elicit both positive and negative responses in athletes, ultimately affecting athletic performance. Different individuals may perceive environmental demands differently, leading to varying stress perceptions and coping responses (Elliott et al., 1994). The affects of perfectionistic climate as a stressor on athletes is not universally harmful, as the extent to which athletes are affected by this stressor varies. The implications of stressors on outcomes depend largely on athletes’ cognitive evaluations of the stressor, their perceived resources available for coping with stress, and their coping strategies.

The Stress Cognitive Appraisal Theory particularly emphasizes individuals’ cognitive appraisal of stressors and divides it into two processes: primary appraisal and secondary appraisal (Lazarus and Folkman, 1984). Athletes’ cognitive appraisal of the perfectionistic climate should also go through these two processes. In the primary appraisal stage, athletes focus on whether and to what extent the perfectionistic climate would affect their well-being. In the secondary appraisal stage, athletes evaluate the characteristics of the stress caused by the perfectionistic climate, consider the feasibility of various coping strategies, the likelihood of potential coping strategies achieving the desired effect, and their own ability to effectively use a certain coping strategy (Lazarus and Folkman, 1984; Folkman et al., 1986). The result of individuals’ appraisal of stressors can impact their subsequent coping strategies (Jiang and Wang, 2022). Coping refers to cognitive and behavioral efforts made by individuals to manage specific external and/or internal demands (Folkman et al., 1986). When athletes’ evaluation indicates that the perfectionistic climate hinders their well-being and they cannot change the harmful and threatening environmental conditions or resources, they are more likely to adopt negative coping strategies, such as avoidance, distancing, selective attention, and so on. On the other hand, when the evaluation result shows that the perfectionistic climate presents challenges that they can overcome through their efforts or by utilizing available resources, individuals are more likely to use adaptive positive coping strategies, such as redefining problems, generating alternative solutions, and evaluating alternative solutions based on costs and benefits. Both coping strategies can be used to alleviate the harm caused by stressors to individuals and are not inherently good or bad, nor are they mutually exclusive. They reflect different effects of different appraisal results on individuals’ responses (Folkman, 1982). However, athletes can positively impact their athletic performance through the mastery path, whereas the harm path negatively affects athletic performance.

The Stress Cognitive Appraisal Theory points out the individual and environmental factors that impact cognitive appraisal and coping strategies (Lazarus and Folkman, 1984). Individual factors impact athletes’ understanding of stressors, which subsequently affects their stress responses and coping efforts. These individual factors mainly include commitment and belief (Jiang and Wang, 2022). Commitment refers to what matters to the individual and holds significance, while belief represents an individual’s confidence in mastering specific situations, both of which impact athletes’ appraisal and coping with the perfectionistic stressor. Moreover, the evaluation and responses of the same individual in different stressful situations may vary, indicating that the context is also an important factor influencing cognitive appraisal and coping strategies (Lazarus and Folkman, 1984). The coach is a key figure responsible for shaping the extent to which athletes experience an environment that is perfectionistic. The role of the coach and specific coaching practices as key sources of pressure to be perfect are also heavily emphasized in theory relating to the development of perfectionism in sport (Appleton and Curran, 2016). The strongest empirical support in this regard is for the role of unrealistic coach expectations and harsh coach criticism with numerous studies showing positive relationships between these coach behaviors and perfectionism in athletes (Sagar and Stoeber, 2009; Gotwals, 2011; Madigan et al., 2019). Aside from traditional mainstream, overt factors such as variance in coaching ability, player talent, and critical in-game decisions, researchers have shown that team dynamics, which is often less perceptible, may also impact performance (Heuzé et al., 2006). Overall, individual and situational factors are interdependent and jointly affects athletes’ cognitive evaluations and coping strategies regarding the perfectionistic climate. These factors act as potential moderators, influencing the strength or direction of the sharp path and harm path.

## 6 Conclusion

Regarding the perfectionistic climate as a stressor in sports settings, if coaches can facilitate athletes to make reasonable cognitive appraisals, the moderate pressure generated by the perfectionistic climate can be perceived as a pleasant and satisfying experience, presenting a positive challenge. This, in turn, leads athletes to cope positively with the pressure, promoting their competitive performance. Conversely, if athletes’ cognitive evaluations of the perfectionistic climate deviate, transforming it into a threatening stressor, it may lead to excessive pressure without effective coping strategies. As a result, athletes may experience adverse reactions, such as depleting potential energy reserves and dysregulating adaptive mechanisms, thereby affecting their competitive performance.

The stressor of the perfectionistic climate in sports settings is not always disadvantageous; if coaches can guide athletes to have reasonable appraisals, stress can be transformed into motivation. On one hand, enhancing athletes’ capacity to handle pressure is fundamental to their ability to cope with competitive stress. Therefore, during training, coaches can expose athletes to continuous stressor stimuli, leading to biological adaptations that improve athletes’ ability to adapt to organizational environments and cope with pressure. Simultaneously, guiding athletes to understand and perceive excessive expectations from their surroundings will facilitate their growth along the mastery path. On the other hand, coaches can help athletes set challenging goals to unleash their potential. By fostering organizational commitment, enhancing athletes’ psychological capital, and boosting self-efficacy, coaches can help athletes break free from self-imposed limitations, leading to greater improvement and better performances in sports.

## 7 Limitations and future

This study measured multiple key variables, including perfectionistic climate, stressors, coping strategies, and athletic performance. While objective evaluations were used for assessing athletic performance, the remaining constructs were measured through subjective self-reports. The results reported by individuals may be influenced by factors such as social desirability bias, coach leadership styles, and impression management. Future research could consider using modern instruments to collect objective data for standardized measurements of elements related to the dual-path model. For instance, when assessing individuals’ cognitive appraisals of stressors, collecting relevant information from training diaries could be employed to measure their stress coping strategies effectively. The use of advanced scientific methods can provide more accurate verification of the relationships between elements of the dual-path model, enabling better application and generalization of the theory in other sports domains.

Additionally, although this study employed multilevel linear growth modeling to analyze the independence of individual-level data within the nested data structure, the data were collected only from core players of the top 24 women’s basketball teams in the 24th edition of CUBAL in 2022. Given the limited number of teams in the study, the absence of nested effects may be specific to this dataset. Future research should consider investigating the influence of individual-level and team-level factors on each other in order to obtain a more comprehensive understanding of the phenomenon. In future research, it may still be necessary to consider the mutual effects between individual-level and team-level factors. Recent research by González-García et al. (2023) has found that improving the quality of coach-athlete relationships, including closeness, commitment, and complementarity, can optimize precompetitive task-oriented coping and the intensity of positive affect before competitions, thereby promoting athlete satisfaction and goal attainment. This also serves as a reminder that individual and situational factors are interdependent and jointly influence athletes’ cognitive evaluations and coping strategies regarding the perfectionistic climate. These factors act as potential moderators, influencing the strength or direction of the sharp path and harm path.

## Data availability statement

The original contributions presented in the study are included in the article/supplementary material, further inquiries can be directed to the corresponding authors.

## Ethics statement

The studies involving humans were approved by the authors hereby declare that all methods were carried out in accordance with relevant guidelines and regulations. The Academic Ethics Committee of the School of Physical Education and Sport, Beijing Normal University, approved the research protocol of this study (BNUCPES0021). Informed consent was provided by the participants. The studies were conducted in accordance with the local legislation and institutional requirements. The participants provided their written informed consent to participate in this study.

## Author contributions

MM: Conceptualization, Formal analysis, Funding acquisition, Investigation, Project administration, Writing – original draft, Writing – review & editing. R-HS: Conceptualization, Data curation, Investigation, Writing – original draft, Writing – review & editing. KK: Conceptualization, Investigation, Writing – original draft, Writing – review & editing. R-RZ: Conceptualization, Formal analysis, Writing – original draft. LC: Conceptualization, Formal analysis, Investigation, Writing – original draft. WW: Conceptualization, Data curation, Investigation, Writing – review & editing. WL: Conceptualization, Writing – review & editing. M-CH: Conceptualization, Data curation, Formal analysis, Writing – review & editing.

## Footnotes

^1^ www.sports.edu.cn

^2^ www.sports.edu.cn/web/notice/images/20222231645585483854_99.pdf

^3^ https://www.douyin.com/

^4^ https://www.xiaohongshu.com/

^5^ https://weibo.com/
